# Colonic Stenting in the Emergency Setting

**DOI:** 10.3390/medicina57040328

**Published:** 2021-04-01

**Authors:** Mario Morino, Alberto Arezzo, Francesca Farnesi, Edoardo Forcignanò

**Affiliations:** Department of Surgical Sciences, University of Torino corso Dogliotti 14, 10126 Torino, Italy; alberto.arezzo@mac.com (A.A.); ffarnesi1@gmail.com (F.F.); edoardo.forcignano@edu.unito.it (E.F.)

**Keywords:** colonic stent, colonic obstruction, colorectal cancer

## Abstract

Nowadays, colorectal cancer (CRC) is the third most frequent cancer, and about a third of patients with CRC presents themselves with symptoms of large bowel obstruction. Historically, surgical resection was the treatment of choice for colonic obstruction, but this kind of approach is burdened by a high risk of postoperative morbidity and mortality. In recent times, the use of a colonic stent has been proposed to overcome the obstruction and transform an emergency surgical case into an elective one to avoid emergency surgery complications. Endoscopic stenting is the first-line treatment option in the palliative management of colonic obstruction, and there is sufficient scientific evidence to support this approach. However, endoscopic stent used as a bridge to surgery is not yet widely adopted because the concern was raised about the long-term survival and cancer safety of this approach. The recent scientific evidence has shown that this approach improves the short-term outcomes, such as postoperative complications and the stoma rate, without differences in long-term outcomes compared to emergency surgery. Therefore, the European Society for Gastrointestinal Endoscopy in 2020 has reconsidered stenting as a bridge to surgery as a valid alternative to emergency surgery.

## 1. Introduction

Globally, colorectal cancer (CRC) is the third most common cancer in males and the second in females, as reported by Globocan in 2018. The incidence decreased in patients older than 50 years, whereas it increased in patients younger than 50. Males are affected more often than females by about 25% [1,2,3]. According to ESMO (European Society for Medical Oncology) guidelines, mortality declined progressively over the last decades, accounting today for 15–20/100,000 in males and 9–14/100,000 in females.

Screening programs for CRC indeed reduced the incidence of advanced diseases. Nevertheless, about 30% of patients affected by colorectal cancer report symptoms of large bowel obstruction. This is notably reported when the lesion is located at the upper rectum or distal colon. This is supposed to be related to the smaller size of the lumen and the increased consistency of the stools [4]. In this manner, 8% to 13% of colon cancer patients develop large bowel obstruction [5]. Intestinal obstruction may lead to various clinical conditions, and it is necessary to quickly and correctly diagnose this condition for setting the appropriate therapeutic algorithm. If not treated promptly, this condition could lead to bowel ischemia, necrosis, and perforation [6,7]. 

The present review aims to assess the role of colonic stenting in the emergency setting according to the latest evidence of the literature and international guidelines.

## 2. Clinical Presentation and Patient Assessment

Patients with bowel obstruction can present with acute abdominal pain, typically cramping with paroxystic episodes every 20–30 min, bloating, obstipation, or chronic and progressive change in bowel habits until failure of passage of the flatus and stools. Nausea and vomiting are more commonly associated with proximal bowel obstruction, provided that the ileocecal valve is incompetent. Not rarely, the competence of the ileocecal valve prevents distension of the ileal tract if not in the late stages. Acute pain in the pelvis with tenesmus is a typical sign of rectal obstruction. Focal pain is usually due to necrosis or ischemia that determine peritoneal irritation. This is generally located in the lower right quadrant and corresponds to the significant dilation of the caecum, particularly in cases of the competent ileocecal valve. In case of considerable distension, the caecum, having the thinner wall of the entire large bowel, is subject to diastasis of the muscular fibers, preluding to perforation. 

An abdominal computer tomography (CT) scan with i.v. contrast completes the patient assessment. This aims to achieve a diagnosis, and in case a neoplasm is assessed, to stage it in order to provide sufficient information for the therapeutic algorithm. In this case, the CT scan should be extended to the examen of the thorax. CT scan with contrast is the standard to confirm the diagnosis because of the high sensitivity and specificity in detecting colorectal cancer [8]. Moreover, it gives the possibility to evaluate the presence of distant metastasis, multifocal disease, carcinomatosis [9]. Therefore, when a neoplasm is confirmed at the abdominal scan, it is always advisable to extend CT to the chest. Up to 20% of newly diagnosed cancers have synchronous metastasis, although most frequently located in the liver [10].

Despite the high sensitivity, it may be argued that a CT scan does not offer high specificity as several misdiagnoses are reported. These entail as high as 15% of the cases, being in about half of the cases diverticular pseudotumors. While no-one would object to a surgical indication in these cases, different is the case of misdiagnoses consisting of Clostridium Difficilis colitis, ischemic colitis, or stool impaction as recently reported. Therefore, it has been questioned if, in all cases, a flexible endoscopy exam should follow a CT scan for confirmation of the diagnosis. However, an agreement on this has not reached.

## 3. Treatment Strategy

First of all, it is necessary to assess if the intent is curative or palliative. According to European Society for Medical Oncology (ESMO) guidelines 2020, it is essential to distinguish among patients with localized colon cancer, patients with oligometastatic disease, and diffuse metastatic disease. For localized colon cancer, TNM staging remains the most relevant criteria for risk assessment. The oligometastatic disease is characterized by metastasis at up to three different sites, with five or more lesions (most of them visceral rather than lymph nodal). Liver metastases could be resected at once or at a second stage, if less than five. An R0 resection should be performed in these cases, provided that more than 30% of liver is left and there is no other concomitant extrahepatic unresectable disease. According to this, any patient with limited liver and/or lung metastases should be considered for potential secondary resection with curative intent. For a patient with diffuse metastatic disease or unresectable disease, the best supportive care, such as palliation, is indicated.

Curative intent can be pursued either by stent bridge-to-surgery (SBTS) strategy followed by elective surgical resection in one stage or two stages, just as palliative intent may be offered more likely with stent placement, if feasible, this way offering the chance of a quick onset of oncologic therapies [5,11]. In both cases, patients’ preferences and thorough discussion should be taken into consideration. This should include the following factors associated with colonic stenting: technical and clinical failure rates, risk of perforation, lower overall complication rates, similar postoperative mortality, higher one-stage surgery and lower stoma rates, potentially higher recurrence rates, similar overall survival, and availability of expertise [5]. 

## 4. Self-Expanding Metal Stents

### 4.1. Stent Choice

Available stents are fully covered, partially covered, and uncovered. The first ones hypothetically reduce tumor ingrowth, but they do not have an excellent grip on the intestinal wall, so they are more at risk of migration. A higher grip is offered by uncovered stents, although they are subjected to tumor ingrowth and, therefore, stent occlusion [12]. Moreover, uncovered stents are indicated as responsible for the tumor’s micro-perforations that are likely to cause the worsening of the prognosis in some patients who receive an SBTS treatment with curative intent. This was the hypothesis at the end of the Stent-in-2 trial [13], which led to the abandonment of the SBTS strategy as recommended in the European Society for Gastrointestinal Endoscopy (ESGE) guidelines which appeared in 2014 [14]. As both the Enteral Stent for Colonic Obstruction (ESCO) trial [15] and the ColoRectal Endoscopic Stenting Trial (CREST) trial [16] did not fully confirm this, ESGE published an update of guidelines in 2020. Here, ESGE recommends that SBTS should be discussed as a treatment option in patients with potentially curable left-sided obstructing colon cancer as an alternative to emergency resection or deviating stoma within a shared decision-making process. When a stent strategy is chosen, an uncovered stent should be preferred for malignant colonic obstruction. This is associated with fewer complications, less tumor overgrowth, less stent migration, longer stent patency, though more tumor ingrowth. Nevertheless, when to prefer a covered or uncovered stent is still open and is currently the object of the CREST 2 trial [ISRCTN54834267]. 

Other stent characteristics are still the object of debate, although there is very limited evidence for a recommendation. Regarding stent diameter, smaller caliber stents have shown less mechanical stress but more risk of migration [17,18]. It is suggested to choose a diameter of more than 24 mm to limit the risk. Regarding stent length, it is recommended to overcome the stenosis by at least 1.5–2 cm on each side [19]. 

### 4.2. Stent Positioning

Although not a standard one, even endoscopic stent positioning requires some bowel preparation. This consists of one or more enemas, which are sufficient to prepare the bowel distal to the obstruction and facilitate the procedure [5]. The belief that if an obstruction is in the act, the distal colon and rectum are necessarily empty is unfortunately not true. Mostly, this is filled with a solid material that could not proceed due to the lack of peristaltic movement [20]. 

Antibiotic prophylaxis is generally unnecessary, although in selected cases, this is suggested [5]. In fact, in the presence of a considerably dilated colon, transient bacteremia due to both insufflation and micro-perforations may occur, although this is not our usual policy [13,21]. 

The severity’s grade of the obstruction might be challenging to assess. Several studies tried to determine it but reported inconsistent outcomes [22,23]. This might influence the rapid opening of the stent and, therefore, the resolution of the obstructed status. It might take several hours before the stent is fully opened, so that it used to be advised to check its correct opening and positioning by X-rays 24 h after the delivery. This was also recommended to check if the obstructed status had evolved positively or negatively. Most importantly, if gas leaks could be observed as air collections in the peritoneal sack as layers below the diaphragm. The relatively rarity of misdiagnosed perforations and their scarce clinical relevance led to abandon this practice as a routine.

Instead, it is a good practice to rinse the stenosis with hydrosoluble contrast enema at the end of the positioning maneuver to detect under fluoroscopy both the patency of the lumen after stenting and the absence of leaks. In any case, it is recommended to not perform stricture dilation due to the increased risk of colonic perforation [24,25]. 

### 4.3. Contraindications

Stents in the colon and rectal tracts can be positioned using either a through the scope or an over the wire technique, although the first one is much more in use as relatively easier. In fact, when the stent is advanced though the scope, this is pushed through the working channel, which makes the handling of the device easier to direct through the stenosis. In both cases, the stent advances over a stiff guidewire with a technical and clinical success rate of 83–100% and 77–100%, respectively [26,27,28,29,30,31]. A higher clinical success rate can be reached by a more experienced endoscopist [13].

In the past, the use of stents in colorectal occlusions had been pointed out as responsible for a worse survival, both overall and disease-free [32]. This was then shown to be dependent on a higher incidence rate of liver metastases, albeit in a retrospective study highly questionable for methodology [33]. Nor was this finding ever confirmed in subsequent studies, especially prospective ones.

Today, the only real contraindication for stenting is the risk of perforation. In a critical appraisal of oncological safety of SBTS in left-sided colon cancer obstruction, Amelung et al. observed that in those studies in which the occurrence of perforation was <8%, the 3-years survival was significantly lower compared to emergency surgery (ES) (OR 0.71, 95%CI 0.52–0.97, *p* = 0.03). Differently, considering those studies in which the occurrence of perforation was >8%, the 3-years survival did not differ compared to ES (OR 1.07, 95%CI 0.76–1.50, *n =* 0.72) [34].

This obviously influences the indication to place a stent in the colon, reserved for obstructed patients. ESGE recommends the placement of a stent only in patients with clinical symptoms and radiological signs of malignant large bowel obstruction, whilst prophylactic treatment is not recommended. 

At the same time, not all the locations of obstruction may be easy to stent. While some sites are relatively easy to reach and stent, some others are challenging. In general, all the junctions between straight tracts of the large bowel may be challenging to approach, particularly in indeed obstructed patients, due to the distention above, which might increase the angle of the lumen bend. This is particularly true at the splenic flexure, which is per se the most angulated tract of the large bowel. Here, it is reported the higher risk of perforation any time the stent has to cross the flexure. Despite the excellent flexibility of the new Nitinol materials, provided they have sufficient expanding force, the stent, which is continuously an object of a straightening force, tend to erode either at the proximal or distal edge of the stent cylinder. Moreover, despite using the most atraumatic hydrophilic guidewires and cannulas, the passage through real tight stenosis located at a natural flexure may put the delivery mechanism with its anvil at increased risk lumen perforation.

A further but relative contraindication is represented by the short distance from the anal verge. Stent placement within 5 cm of the anal verge is usually avoided because of the possibility of severe pain, tenesmus, and rectal bleeding. However, some patients who wish to prevent an ostomy can undergo stent placement very low in the rectum with good tolerance. In these cases, always with palliative intent, the maneuver may be attempted as it would be easily reversible in case of intolerance.

### 4.4. Complications and Their Management

The worst complication it can occur while stenting an obstructed colon is perforation. A systematic review shows a median perforation rate of 4.5% [35]. To reduce this kind of risk, it helps minimize the insufflation, and it is suggested to choose carbon-dioxide insufflation. Perforation could be detected at the time the stent is positioned or later. Early perforation may occur while advancing the guidewire or cannula, or while advancing the stent’s anvil, or it could be due to excessive insufflation to achieve bowel distention through the entire maneuver. Delayed perforation may occur due to prolonged decubitus of the stent, which could happen at an interval of at least 15 days before elective surgery. Moreover, as mentioned above, some studies report that perforation could lead to peritoneal seeding and worse oncological outcome [36]. In all cases, ES is recommended in these cases.

Migration happens in approximately 10% of stent positioning maneuvers [35], although less when uncovered stents and stents larger than 24 mm in diameter are employed. Late migration could also be due to the downstaging of the lesion after chemotherapy. The palliative setting can be treated with stent’s replacement or stent-in-stent techniques showing immediate clinical success in 75% of the patient and the median duration of stent patency of 170 days [37,38]. On the other hand, in the SBTS setting, patients with stent migration undergo earlier surgery. 

Re-obstruction happens almost exclusively in the palliative setting. It helps position the stent exceeding 2 cm from the tumor proximal and distal margin to reduce this risk. 

## 5. The Curative Scenario 

Two different strategies are possible in the emergency setting to manage malignant colonic obstruction if a curative intent might be pursued. The first is represented by ES, including various options, from the simple diverting stoma to the subtotal colectomy, including Hartmann procedure (HP) and intraoperative wash-out with primary anastomosis with or without diverting stoma. Some of them require a second treatment to close a stoma or resect the primary tumor if a simple stoma had been performed initially. In a few cases, even a third surgical procedure is required. It is difficult to restrict the options available in the emergency setting and design a prospective randomized trial that would be very much awaited to clarify the surgical outcomes of the different procedures and the functional outcomes, less critical but still absolutely relevant in this context. Indeed, the patient’s age, his/her willing, and his/her life expectations are of paramount importance to balance the benefits of the different options. In this scenario, the possibility to return to an elective treatment condition would play a significant role. This is now again offered by the chance of stenting malignancies resolving the obstruction, delaying the treatment to election. It is, therefore, quite challenging to drive the indisputable and incontrovertible conclusion. 

### Stent as a Bridge to Surgery Versus Emergency Surgery

In recent times, the results of two randomized controlled trial studies comparing SBTS versus ES became available: ESCO study [15] and CREST study [16]. The first one analyzes 144 patients randomly assigned to undergo SBTS or ES with a minimum follow up of 36 months. It shows a lower median operating time, a higher number of resections performed laparoscopically with a higher rate of primary anastomosis and a more extensive lymphadenectomy in the first group with a 100% of R0 resection rate without a difference in disease-free survival and overall survival. The second one analyzed 246 subjects enrolled in 39 different centers. It shows a lower stoma formation rate in the SBTS subgroup, with no difference in 30 days postoperative mortality, length of hospital stay, and quality of life. Nevertheless, it shows no difference in 3 years mortality between the two treatment groups. 

In a meta-analysis of RCT’s of our conception [39], SBTS seems to guarantee a lower rate of diverting stoma both in the emergency and in the elective subset and to be considered a safe short-term technique. Concerns have been expressed about oncological outcomes, but more recent international guidelines [40,41] suggest that SBTS is a possible alternative to ES. The latest ESGE guidelines update appeared in the literature in 2020 recommending SBTS “to be discussed, within a shared decision-making process, as a treatment option in patients with potentially curable left-sided obstructing colon cancer as an alternative to emergency resection. This discussion should include the following factors: availability of required stenting expertise, risk of stent-related perforation, higher recurrence rates, similar overall survival and postoperative mortality, lower overall complication rates and permanent stoma rates, a higher proportion of laparoscopic one-stage surgery procedures, and technical and clinical failure rates of stenting” with a strong recommendation and high-quality evidence.

Nevertheless, at the time of the embargo for colonic stents, particularly in the Netherlands, the policy of performing a simple diverting stoma in an emergency became popular. This had the advantage to delay the proper colorectal resection to a later time in the hands of a more expert and dedicated colorectal surgeon. So far, only a few studies have compared SBTS with diverting stoma (D, S), and none of them are randomized. Amelung et al. [38] show evidence favoring SBTS, a lower rate of a temporary stoma, surgery, and long-term complications with no difference in terms of mortality and morbidity, disease-free survival, and overall survival. On the contrary, Mege et al. [42] show better short-term outcomes for SBTS, the same median disease-free survival but a higher overall median survival in the DS group. It has to be observed that the latest finding is likely to be due to the high rate of stent perforation in the study by Mege (11% vs. 1.9% in the group analyzed by Amelung). 

Sensitivity analysis has shown that experience, techniques, and case volume might influence long-term outcomes and treatment decisions [35]. ESGE guidelines recommend the construction of decompressing stoma if the patient is not a candidate for colonic stenting or if experienced endoscopists are not available, but useful quality data are lacking. 

## 6. The Palliative Scenario 

Palliative stenting for malignant colonic obstruction has been proven effective and safe as a first-line treatment when not contraindicated. In this setting emerged that stenting, compared with ES, results in shorter hospitalization, lower intensive care unit admission rate, shorter time to start chemotherapy, significant lower incidence of stoma formation, resulting in an increased quality of life, even if the clinical relief of the obstruction is greater in the patients treated by emergency surgery [43,44,45,46]. Thus, ESGE, in the latest guidelines, strongly recommend, with high-quality evidence, colonic stenting as the preferred treatment for palliation of malignant colonic obstruction. 

## 7. Right Colonic Obstruction

A systematic review published by Amelung et al. [47] compared SBTS and ES for malignant obstruction of the proximal colon (MOPC). Primary resection and anastomosis represent the current standard of care for the treatment of MOPC, chosen in 94.8% of the systematic review patient. However, this is burdened by a higher mortality rate of ES resection compared to elective surgery. This confirms the needed for alternative treatment options. STBS has been performed in 1.3% of patients. It showed a lower mortality rate, a lower risk of anastomotic leak, a lower rate of permanent stoma creation, and overall a lower rate of major complications. So, ESGE recommends considering colonic stenting for malignant obstruction of the proximal colon both as a bridge to surgery or as a palliative means, although with weak strength of recommendation and low-quality evidence. 

A more recent systematic review published by Boeding et al. [48], published after the release of ESGE guidelines, suggests an advantage of sequential treatment over ES resection, especially for short term outcomes. More complications were described after ES. Nevertheless, it has to be noticed that the authors declared a selection bias, as patients with sepsis or unstable vital signs were excluded from the SBTS treatment but not from the analysis. Moreover, up to 23.2% of ES patients had stomas created, whereas no patients treated with SBTS treatment required a stoma. Long term outcomes appear to be comparable. Stenting positioning was technically successful in 84–96% of patients.

## 8. Conclusions

Colorectal stenting, with endoscopic deployment, is a relatively new technology that might be useful in an emergency, such as colonic obstruction caused by primary colorectal cancer. It can quickly resolve the occlusion by allowing the passage of stools and gases, improving the clinical condition of the patient. Therefore, stenting may be used as an alternative to emergency surgery in the palliative setting. At the same time, in the curative setting, it can be used in a sequential bridge to surgery policy. While there have been concerns about long-term oncological outcomes of SBTS, the more recent scientific evidence suggests that this kind of approach is comparable with ES in terms of oncological results.

## Data Availability

Not applicable.
